# Discrimination and technology-based disease control

**DOI:** 10.1371/journal.pone.0357004

**Published:** 2026-09-23

**Authors:** Marion C. Aichberger, Karolina Fetz, Suyi Leong, Heejung S. Kim, David K. Sherman, Tim S. Müller

**Affiliations:** 1 Faculty of Humanities and Social Sciences, Berlin Institute for Integration and Migration Research, Humboldt-Universität zu Berlin, Berlin, Germany; 2 Department of Psychiatry and Psychotherapy, Division of Social Psychiatry, Medical University Vienna, Vienna, Austria; 3 Department of Psychological and Brain Sciences, University of California, Santa Barbara, California, United States of America; PLOS: Public Library of Science, UNITED KINGDOM OF GREAT BRITAIN AND NORTHERN IRELAND

## Abstract

**Objectives:**

Experiences of ethnic discrimination may undermine compliance with public mitigation policies for disease control, such as Digital Contact Tracing (DCT), through increasing concerns, undermining trust and decreasing solidarity.

**Methods:**

Addressing this issue, we collected a web-based quota sample of the German population (N = 700) in September 2020 focusing on public health strategies aimed to curb the spread of SARS-CoV-2.

**Results:**

Using a series of mediation models employing structural equation modelling we indeed find evidence that discrimination experiences, mediated via privacy concerns and trust in government, negatively predict DCT support and DCT opt-in. Our data, however, do not support the mediating role of solidarity. Additionally, we find that among individuals who were not using DCT, discrimination experiences were negatively associated with people’s willingness to participate in DCT in the future. This relationship was mediated by concerns that the government might use DCT against one’s ethnic group.

**Conclusion:**

Experiences of ethnic discrimination can decrease the effectiveness of health policies including respondent-driven tools like DCT, as their support depends strongly on the trust in government. Current and future policies should take ethnic minority concerns into account in order to improve their widespread acceptance.

## Introduction

The rapid improvement of information and communication technologies (ICT) have led to an increasing exploration and development of technology-based monitoring, surveillance and prevention methods to complement existing strategies for disease control, in particular infectious diseases [1]. Strategies for early warning and adaptive response system (EWARS) for highly contagious diseases [2], such as Dengue [3] or Ebola [4] are promising developments which are envisioned to reduce their spread. While attitudes towards respondent-driven tools, such as needed for digital contact tracing (DCT), may be acceptable in the general population [5], the use of sensitive personal information for an automated technological approach also warrants the evaluation of individual and group-based barriers to its acceptance.

The COVID-19 pandemic has caused more than 6.9 million deaths worldwide [6]. Initially, limited treatment options for COVID-19 and the non-existence of vaccination against the virus [7] made effective non-pharmacological mitigation strategies to reduce community transmission a priority for public health authorities, thus lending it as a valuable target for evaluation of the acceptance of a respondent-driven tool in disease control in a real-life scenario. While vaccinations and a change of the virus’s pathogenic structure slowed down its spread and ended the global pandemic emergency, the possibility of future outbreaks of SARS-CoV-2 or other zoonotic diseases remains uncertain. Since future pandemics are still likely to occur, the use and improvement of non-pharmacological mitigation strategies, in particular ICT tools [8], in the future will probably still be necessary.

Regardless of the mode of transmission, contact tracing, as one of such mitigation strategies, has long been a cornerstone of communicable diseases prevention [9]. It has also proven to be effective during the COVID-19 pandemic [10]. However, personal contact tracing is not only time-consuming and needs large numbers of personnel, but disclosing one’s personal contact history to a stranger may also be seen as invasive [11]. Throughout the pandemic a strong focus was thus placed on using digital solutions, i.e., Digital Contact Tracing (DCT) [12]. DCT apps employ smartphones’ Bluetooth signal to detect when two app users are close to each other for a prespecified duration and distance. Users are notified if a previous contact tests positive, and are eligible to be tested and quarantine. DCT represents a relatively low-cost intervention from the perspective of the individual and might mitigate infection spread substantially, by reducing the time required to isolate infected individuals and their contacts [13]. In view of this, digital solutions for contact tracing in form of mobile phone applications have been developed [14]. Yet, for DCT to be effective, a critical threshold of DCT users is needed. Indeed, it was estimated that for DCT to be an effective means in spread of SARS-CoV-2 at least 60 percent [15], or even up to 96% of the population would have to have used the app [16,17]. Hence, it is crucial to uncover any potential hindrances to the acceptance and implementation of DCT as a non-pharmacological approach for curbing virus transmission.

The success of even a seemingly simple intervention such as DCT might depend on people’s privacy and related concerns and build upon individuals’ trust and sense of (perceived) solidarity. If such concerns are present or trust and solidarity have been eroded, a widespread adoption of DCT cannot be expected. Another precursory factor, which has been overlooked in previous research, is ethnic or racialized discrimination. Examples of direct and overt forms are name calling, housing or labour market discrimination, which might increase concerns and an erosion of trust among those who have had to endure it [18]. These experiences can therefore reduce the willingness to participate in DCT. This study therefore seeks to illuminate these barriers to DCT adoption in the general population by unpacking the effect of perceived ethnic discrimination into various potential pathways through which it might reduce DCT adoption. We utilize an oversample of persons with an immigrant background in Germany, that allows us to test these mediation effects with sufficient statistical power. The study therefore highlights particular concerns among DCT-non-users, and holds important implications for infectious disease mitigation strategies and future pandemics.

### Privacy concerns, trust, and solidarity as central determinants of DCT up-take

Previous research has identified a number of factors that can affect DCT acceptance to curb the spread of Coronavirus, for example belief in the effectiveness of precautions [19–21] or one’s own perceived vulnerability towards COVID-19 [22,23]. In contrast, fears over data privacy have been identified as one of the strongest barriers for the use of DCT [24–28].

Another crucial predictor for DCT use is trust in the government or health authorities (for DCT use: [21,22,24], for other protective behaviors:[29–31]). Indeed, trust in the quality and efficacy of the political system and its institutions and representatives, has been shown to be essential for collective action, in general, to succeed [32]. Low trust levels, in contrast, may therefore hinder such action [33]. In fact, early investigations on DCT acceptance, suggest that individuals with limited trust in their national government were also more reluctant to install and use contact tracing apps [22,24].

In addition to beliefs about the government, people may also be influenced by their beliefs about their fellow citizens, and in particular, whether they develop a sense of solidarity. While some studies indicate that compliance with COVID-19 prevention measures was to a certain extent driven by a self-protective motivation [34] and the importance of one’s own health [29], it can also be conceptualized as an act of solidarity. Solidarity, in the conception of Hechter [35], can be broadly defined as the extent to which members of a group are following an obligation to contribute their individual resources (also compliance with measures, time, wealth etc.) to the goals of that group without receiving any individual compensation. These group goals often entail the provision of collective goods. A stronger definition, emphasizing prosocial concerns, would be the “willingness to make sacrifices for the wellbeing of the other members of a group” (Beckert as cited by Trampusch: [36]). While a participation in DCT might not be a “sacrifice” it is still an individual contribution that can incur certain costs, e.g., giving up some privacy. Depending on the way that DCT is set up in different contexts, gaining information about potential risk encounters might be seen as a form of individual compensation. Prosocial concerns and the desire to protect close persons have indeed been identified as important factors increasing the likelihood of DCT use and adherence to other preventive measures [24,37].

Overall, these findings suggest that privacy concerns can undermine, while trust and solidarity can increase people’s likelihood to comply with DCT to curb the spread of viruses such as SARS-CoV-2. Yet, as will be argued in the following, an important determinant that can increase individuals’ privacy concerns and erode their trust and solidarity and thereby hamper DCT compliance is ethnic discrimination.

### The role of ethnic discrimination for the formation of trust, privacy concerns, and solidarity

The effectiveness of technologies such as DCT is highly dependent on a high uptake in the general population [38]. However, studies on health behaviour and engagement in prevention efforts (see for example [39,40], for Germany: [41]) may suggest that racialized minorities and migrants may be more reluctant to participate in public health measures. Also, data from the COVID pandemic pointed towards a lower uptake of vaccinations in groups such as racialized minorities, refugees and migrants [42] as well as lower willingness to participate in measures like DCT [43–46]. Lower levels of trust in the (health care) system and experiences of discrimination have been suggested to be relevant factors for this behaviour (see, e.g., [47,48]). As trust in government is particularly central to the willingness to accept measures suggested by such, and racialized minorities may be reasonably and particularly apprehensive about government, we examined perceived discrimination as another factor that may underlie resistance to participate in DCT by undermining trust and solidarity.

First, ethnic discrimination has indeed been found to negatively impact preventive behavior in racialized groups [49–52], which in part has been shown to be mediated by trust [53,54]. Furthermore, discrimination is frequently experienced by racialized groups accessing health care [48,55–59] and has also been associated with diminished trust in the health-care system [60–62], delays in seeking treatment [40,63], and lower adherence to a physician’s recommendations [64]. Moreover, inequality erodes trust levels in diverse communities. Discrimination and prejudiced media coverage have been associated with decreased trust in the government over time, particularly in racialized groups [65–67]. However, the effects of discrimination on trust among racialized groups are complex and might vary between different groups, immigrant generations, and societal contexts. Additionally, different kinds of trust are affected differently in the face of discrimination. For instance, People of Color in Canada experience higher levels of ethnic discrimination, which contributes to a gap in generalized trust and trust in specific others compared to the majority population [68]. Similarly, among ethnic minority immigrant populations in Europe, self-reported discrimination is a strong negative predictor of trust in public institutions [69]. Taken together, discrimination overall emerges as a factor that undermines trust. These potential group differences non-withstanding, we will focus on perceived discrimination experiences as a predictor, since the objective belonging to a potentially heterogeneous immigrant group might have less clear effects than perceived discrimination, which has been well-established for a broad range of health behaviors (see above).

Second, having experienced ethnic discrimination may also diminish willingness to participate in DCT by increasing privacy concerns among racialized groups. Also, more recent reports of heightened mistrust towards contact tracing methods during the COVID-19 pandemic within the BAME (Black, Asian and Minority Ethnic) and BIPOC (Black, Indigenous, and People of Color) communities in the UK and US [70,71] point towards an increased sensitivity regarding automated means for surveillance in racialized groups. It might induce worries about being ‘exposed’ as carrier of the virus and that this might be attributed to one’s ethnic group membership in a stigmatizing way. Also, it might induce fears of abuse of DCT infrastructures for later surveillance purposes targeted against one’s group. Indeed disconcerting in this respect are concerns surrounding the potential misuse of public health data, fueled by incidents such as Minnesota law enforcement describing their investigative profiling of Black Lives Matter protesters as ‘contact tracing’ in May 2020 [72], or by increasing the risk of involuntary ‘outing’ among visitors after a cluster outbreak in Seoul’s gay nightlife district [73].

Third, ethnic discrimination might also hamper DCT use by undermining people’s solidarity. Twenge et al. (2007) notes: “Prosocial behavior depends on believing that one is part of a community in which people mutually seek to aid [and] to support […] each other. […] When people feel excluded, their inclination to perform such behaviors should be reduced or eliminated” [74], p 56). Subjecting people to social exclusion does indeed lead to a reduction of their prosocial behavior, mediated via the reduction of empathetic concerns for others [74]. Therefore, ethnic discrimination may reduce feelings of solidarity in general or people might fear that their own solidarity towards others might not be reciprocated. (Note that we understand solidarity here broadly, not related to any subgroup in particular. This is also reflected in our choice of measurement, described further below.) We therefore include measures of solidarity in our study that take into account not only whether people express concerns for the wellbeing of other groups in society, but also whether they perceive solidarity being extended towards them. We further test whether these perceptions of solidarity affect the willingness to participate in DCT.

### Aim and hypotheses of the study

While the majority of studies examining indicators of DCT app use include privacy concerns, government trust and vulnerability as central predictors, this is to our knowledge the first study to examine how ethnic discrimination affects attitudes towards participation in DCT via different pathways. We hypothesize that individuals who experience higher levels of ethnic discrimination will be less likely to support DCT policies and opt-in to DCT, as they might (1) experience stronger privacy concerns (Privacy pathway), (2) be less trusting towards the government or other persons in general (Trust pathway) and (3) might hold doubts that solidarity will be extended towards them or show less solidarity for other reasons (Solidarity pathway).

We conceptualize privacy concerns in a broad way, including concerns regarding personal data (general privacy concerns), concerns about one’s reputation that is linked to personal information (reputation concerns) as well as the potential use of personal information against oneself or one’s social or ethnic group (relational concerns). The trust pathway will be examined using two concepts of trust, i.e., generalized trust (i.e., trust towards people in general) and trust in the government. The solidarity pathway will be evaluated addressing two different aspects, i.e., people’s own prosocial concerns for different groups in society (solidarity) as well as people’s perception that other groups in society have prosocial concerns towards their own group (perceived reciprocated solidarity).

Overall, we expect a total negative effect of perceived ethnic discrimination on DCT-support that will be partially or fully mediated via the three hypothesized pathways.

## Materials and methods

### Background on the implementation of DCT in Germany (*Corona-Warn-App*)

Germany chose the development and implementation of the decentralized – privacy-first – approach for DCT, without tracking of GPS data, by phones only exchanging anonymized codes via bluetooth. The Corona-Warn-App used a framework developed jointly by Google and Apple and was developed in a joint approach with SAP and Deutsche Telekom among others, and was official launched mid-June 2020. To ensures transparency the development of the program code was continuously visible on the GitHub platform. Registered users were informed anonymously if they had a risk contact and advised to perform a SARS-CoV-2 antigen test, which was free of charge in these cases.

### Data

The data was collected in an online survey which was administered in Germany in September 2020 (recruitment period: 1 September 2020 until 11 September 2020), and is a part of a multi-national study [75]. However, the specific module on ethnic discrimination experiences was designed exclusively for the German context. Consequently, direct cross-national comparisons regarding the discrimination pathway are not possible within this dataset. Respondents were recruited through an online access panel and were compensated for participating by collecting points, which they can exchange for vouchers, money, or donations. All participants provided written informed consent via an online form prior to their participation in this study. The study was approved by the ethics board of the university of the corresponding authors of this study. The target sample size was *n* = 700 respondents, including an oversampling of respondents with a migration background of n = 200. Migration background was defined on the basis of citizenship, with persons themselves or one of their parents being born with a nationality other than German. The original sample (N = 500) also included 69 persons (14%) with immigrant background to which the boost sample (N = 200) was added. The sample is a quota sample, representative of the German population in terms of age, sex, immigrant background and place of residence^.^ Three cases, identifying as gender diverse, had to be excluded, because for these individuals the design weights used in the present analyses (see below) could not be calculated. The final analysis sample thus comprised *N* = 697 participants.

### Outcome variables

***DCT support*** Respondent’s support for DCT was measured by seven items on a 1 (*strongly disagree*) to 6 (*strongly agree*) scale, which were averaged to create a composite score (*M* = 3.77, *SD* = 1.32; *α* = 0.92). An example item is: “I think digital contact tracing is a good tool to stop the spread of Coronavirus (COVID-19)”.

#### DCT opt-in/future opt-in.

Respondents’ current participation in DCT was measured by one item: “Did you opt-in to Germany’s digital contact tracing app (‘Corona-Warn-App’)?” (yes/no). Only for respondents who reported no current participation in DCT, intentions about DCT opt-in in the future were assessed (“Would you consider opting in to digital contact tracing?” – I am open to opting-in/I will never opt in).

### Main predictor and mediator variables

#### Discrimination experiences.

Perceived discrimination on ethnic grounds was assessed by the following item, measured on a 1 (*strongly disagree*) to 7 (*strongly agree*) scale: “I experience racist discrimination in everyday life, e.g., because I belong to a different ethnic group, due to my foreign nationality or because of my non-White skin color.” (*M* = 1.78, *SD* = 1.38).

#### Privacy concerns (mediator).

**General privacy concerns.** Respondents’ general privacy concerns were measured with four items, each measured on a 1 (*strongly disagree*) to 7 (*strongly agree*) scale, and aggregated into a mean score. An example item is: “I am concerned that unauthorized people may access my personal information.” (*M* = 5.13, *SD* = 1.32; *α* = 0.91).

**Reputation concerns.** Reputation concerns were measured by two items on a 1 (*strongly disagree*) to 7 (*strongly agree)* scale that were averaged for analysis. Example: “I would be embarrassed to share certain aspects of my life with people I am close to”. (*M* = 4.43, *SD* = 1.42; *α* = 0.70; r = 0.54, p < 0.001). A factor analysis (PCF) was performed to test whether general privacy concerns and reputation concerns might represent one underlying construct. The analysis yielded a two-factor solution, confirming the creation of two scales.

**Relational concerns (concerns related to the misuse of personal data).** For respondents who are currently not taking part in DCT, we also measured to what extent preferences for not revealing information and concerns about the potential misuse of the data that might (be suspected to) be collected via DCT would constitute barriers to the future opt-in to DCT. In total, four items were used, employing a 1 (*strongly disagree*) to 6 (*strongly agree)* scale, which were introduced into the analysis separately. Two items (‘Government hide’ (*M* = 2.77, *SD* = 1.64) and ‘People hide’ (*M*=2.73, *SD*=1.58)) assessed respondents’ preferences to not reveal personal information to the government or in close relationships, respectively, as reason for them to not participate in DCT (“I don’t want to opt in to digital contact tracing because there are aspects of my life that I do not want the government [people in my life] to know about.”). Two items (‘Government against’ (*M*=2.71, *SD*=1.65) and ‘People against’ (*M*=2.74, *SD*=1.60)) measured respondents’ fear that the information revealed might be used against their ethnic or social group either by the government or by other people as a reason for not opting in to DCT (Item wording: ”I don’t want to opt in to digital contact tracing because the government [other people] might use the information against my ethnic or social community (e.g., tracking and policing during protests) [e.g., exclusion from public places]”).

### Trust (mediator)

**Government trust.** Trust in government was measured with four items, adapted from in the US National Election Study [76] a 1 (*strongly disagree*) to 6 (*strongly agree)* scale and averaged to form a scale (*M* = 3.27, *SD* = 1.13; *α* = 0.85). An example item is: “Most of the time I think I can trust the government to do what is right”.

**Generalized trust.** We used a standard three-item measure to assess respondents’ generalized trust, adapted from Dinesen [77] An example item is: “Generally speaking would you say that most people can be trusted, or that you can’t be too careful in dealing with people?”. Responses were measured on a 0 *(You can’t be too careful)* to 10 *(Most people can be trusted)* scale resulting in a single mean score (*M* = 4.68, *SD* = 1.93; *α* = 0.81)

### Solidarity and perceived reciprocated solidarity (mediator)

We used two scales in order to assess respondents’ solidarity and perceived reciprocated solidarity, each comprising three items. The solidarity measure (adapted with minor changes from the European Values Survey [78]) assesses on a 1 *(very much)* to 5 *(not at all)* scale to what extent individuals feel concerned about the living conditions of people in their neighborhood, people of the region they live in, and their fellow countrymen” (*M* = 3.33, *SD* = 0.81, *α* = 0.86). For the reciprocated solidarity measure, the item wording was reversed: “And now thinking about it the other way around, to what extent do people in your neighborhood [people of the region your live in; your fellow countrymen] feel concerned about the living conditions of your group?” (*M* = 3.06, *SD* = 0.81; *α* = 0.89). Items were reverse-scored and averaged so that higher scores reflect higher levels of solidarity/perceived reciprocated solidarity.

### Control variables

Throughout all analyses we controlled for potentially confounding variables, i.e., political orientation, education level, gender, age, immigrant background, respondents’ state of residence, perceived own vulnerability/ vulnerability of a close person towards COVID-19. As a robustness check, we also ran the models without these covariates in models with standard confidence intervals. The pattern of results stayed the same.

For the analyses all continuous outcome and predictor variables were z-standardized, so the effects between these variables refer to a one standard deviation change. For the DCT opt-in variables the effects can be interpreted as changes in probability of opting in. The descriptive statistics of all variables are provided in Table 1.

**Table 1:** Descriptive statistics.

	Total		No immigrant background		Immigrant background	
	Mean	Std. Dev.	Mean	Std. Dev.	Mean	Std. Dev.
*Dependent variables (range)*						
DCT support (1-6)	3.77	1.31	3.77	1.29	3.76	1.37
DCT opt-in (0-1)	0.39	0.49	0.39	0.49	0.38	0.49
DCT future opt-in (0-1)	0.37	0.48	0.37	0.48	0.38	0.49
*Independent variables*						
Perceived ethnic/racial discrimination (1-7)	1.69	1.30	1.50	1.06	2.23	1.70
Individual vulnerability towards COVID-19 (1-6)	2.92	0.96	2.93	0.95	2.89	1.01
Vulnerability of close person towards COVID-19 (1-6)	3.21	0.99	3.20	0.96	3.22	1.07
General privacy concerns (1-7)	5.13	1.32	5.13	1.32	5.14	1.34
Reputation concerns (1-7)	4.43	1.42	4.44	1.40	4.43	1.49
*Relational concerns*						
Reason no DCT: No information to government (GovHide) (1-6)	2.77	1.64	2.76	1.62	2.82	1.69
Reason no DCT: No information to people that are close (PeopleHide) (1-6)	2.73	1.58	2.72	1.56	2.77	1.63
Fear government uses information against ethnic/social group (GovAgainst) (1-6)	2.71	1.65	2.70	1.63	2.73	1.69
Fear other people use information against ethnic/social group (PeopleAgainst) (1-6)	2.74	1.60	2.74	1.58	2.76	1.65
*Trust and Solidarity*						
Trust in government (1-6)	3.27	1.13	3.26	1.13	3.27	1.12
Generalized trust (0-11)	4.68	1.93	4.70	1.88	4.63	2.07
Solidarity (1-5)	3.33	0.81	3.34	0.80	3.31	0.84
Perceived reciprocated solidarity (1-5)	3.06	0.81	3.09	0.79	3.00	0.86
*Sociodemographic variables*						
Political orientation (left-right scale) (1-11)	5.56	1.95	5.58	1.93	5.51	2.04
Education level						
Lowest/no degree (0-1)	0.11	0.31	0.12	0.32	0.07	0.25
Middle school (0-1)	0.30	0.46	0.32	0.47	0.26	0.44
High school (0-1)	0.29	0.45	0.28	0.45	0.31	0.46
University (0-1)	0.29	0.45	0.27	0.44	0.35	0.48
Still studying/other (0-1)	0.01	0.11	0.01	0.10	0.02	0.12
Gender male (0-1)	0.50	0.50	0.51	0.50	0.46	0.50
Immigrant background (0-1)	0.26	0.44	--	--	--	--
Age (18-90)	44.84	15.47	45.46	15.11	43.09	16.38

Notes: N = 697 (non-immigrant background: 429; immigrant background: 268), except for ‘DCT future opt-in‘ (N = 425; non-immigrant background: 260, immigrant background: 265); design weights have been applied. Dummy variables for federal states have been included in models, but are not shown in table.

### Statistical analysis

We test our hypotheses by estimating multiple mediator models [79] within the framework of structural equation modelling (Stata 15, sem command; StataCorp, [80]). Since we oversampled respondents with immigrant background, design weights were applied so that the analysis sample would represent the population distribution regarding gender, age, immigrant background and state of residence in Germany. All models used robust standard errors, since homoscedastic standard errors cannot be assumed when using survey weights. However, the total indirect effects were tested via bootstrapping (see below). We report the Standardized Root Mean Square Residual (SRMR) as well as the Coefficient of Determination (CD, similar to R-squared) as Goodness-of-fit (GOF) indicators suitable for weighted samples (according to Hu and Bentler [81]. An SRMR close to or below 0.08 is considered a good fit. For the Coefficient of Determination values closer to 1 indicate a good fit. For all mediator variables we relaxed the assumption that their error terms are uncorrelated to each other, estimating covariance parameters between them. All models included the above-mentioned control variables. In order to estimate the indirect (mediated) effect of ethnic discrimination on the outcome variables we applied the Preacher-Hayes method [82]. Hereby, indirect effects were tested via bootstrapping (5000 replications) and considered statistically significant when the percentile-based 95%-confidence interval did not contain 0.

## Results

At the time of data collection (September 2020), 39% of respondents in our sample reported using DCT (June 2020). Overall, 22% of respondents (37% among current non-users) indicated that they would consider opting-in in the future. However, 39% of the overall sample responded that they were neither taking part in DCT now, nor would they consider doing so in the future. Among the bivariate correlations (Table 2), we find several relationships confirmed that have been reported in the literature before. As expected, general privacy concerns and reputation concerns hamper individuals’ support or adoption of DCT. But these effects are smaller than the bivariate effects of perceived vulnerability and trust. Government trust is the strongest predictor; for all bivariate correlations see Table 1. Generalized trust, solidarity and perceived reciprocated solidarity all show positive, but fairly weak relationships with DCT support and use.

**Table 2:** Pairwise correlations (Pearson’s r) between main explanatory variables and outcomes among the full sample.

	1	2	3	4	5	6	7	8	9	10	11	12	13	14	15	16
1 DCT support	1.00															
2 DCT opt-in	.62***	1.00														
3 Ethnic discrimination	−.12***	−.05	1.00													
4 Own vulnerability	.30***	.16***	.08*	1.00												
5 Other’s vulnerability	.26***	.16***	.05	.59***	1.00											
6 Privacy concerns	−.22***	−.15***	.06	.10**	.08*	1.00										
7 Reputation concerns	−.11 ***	−.08*	.05	.10**	−.01	.38***	1.00									
8 Government trust	.58***	.33***	−.11***	.23***	.22***	−.15***	−.06	1.00								
9 Generalized trust	.18***	.15***	−.09**	−.03	−.04	−.17***	−.14***	.30***	1.00							
10 Solidarity	.09**	.09*	−.03	.12***	.11***	.08*	−.03	.18***	.27***	1.00						
11 Reversesolidarity	.11***	.11***	−.06	.08*	.07	−.06	−.02	.22***	.31***	.69***	1.00					
12 Left-right scale	−.17***	.10**	−.07	−.08*	−.08*	.02	.11***	−.18***	−.12***	−.05	−.05	1.00				
13 Education yrs.	.09**	.01	.05	.05	.13***	−.02	−.02	.03	.01	−.05	−.05	−.08*	1.00			
14 Male	.04	.12	.00	.02	−.12***	−.06	.09**	−.04	.08*	.01	.08*	.05	.05	1.00		
15 Immigrant background	.00	−.02	.25***	−.02	.01	.00	.00	.00	−.02	−.01	−.05	−.02	.05	−.07	1.00	
16 Age	.07	.06	−.13***	.33***	.00	.11***	.01	.07	.08*	.18***	.11***	.01	−.03	.12***	−.07	1.00

*Notes.* Correlation coefficients are Pearson’s r with exception of the correlations between the categorical variables DCT-opt in [2], male [14], and immigrant background [15]. Here Tetrachoric correlations are given. The correlation coefficients between the categorical variables and the continuous variables are Point-Biserial correlations. Numbers refer to complete analysis sample (N = 697) and do not contain variables that were only used in the sample of DCT non-users. Variable ‘Education years’ only used for this table, the full analysis uses education degrees (categorical variable). Survey weights have been applied. Significance levels: *** p < 0.001, ** p < 0.01, * p < 0.05

Another important exploratory observation is that respondents with an immigrant background do experience higher levels of discrimination (*r*(695) = .25, *p* < 0.001; non-immigrant respondents *M* = 1.50, *SD* = 1.05; immigrant respondents *M* = 2.21. *SD* = 1.69; mean difference: 0.71; *t*(695) = 6.81, *p* < 0.001). Ye*t* apart from that, no other significant differences between respondents with and without an immigrant background are found. Thus, respondents’ immigrant background is not predictive of DCT support or DCT opt-in, whereas experienced ethnic discrimination is predictive of DCT support (r(695) = −.12, *p* < 0.001) or opt-in. These findings align with previous results that have shown perceived ethnic discrimination to be an important predictor of health-related behaviors [49–51,54]. In the following section, we will examine the theorized psychological mediators to explain the negative relationship between ethnic discrimination and DCT support.

## Mediation analyses

### DCT support

**Fig 1:**
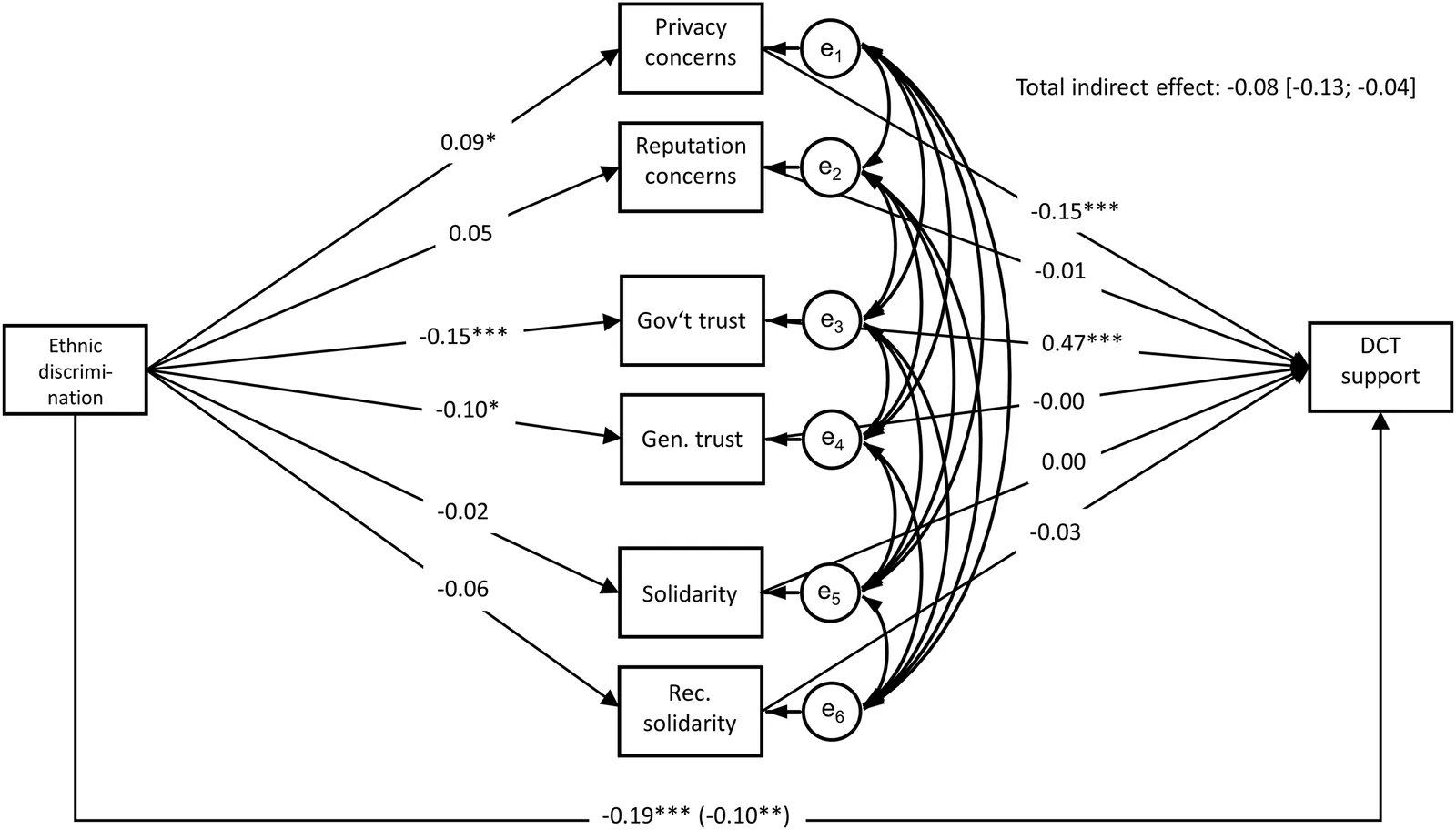
Effect of perceived ethnic discrimination on DCT support, mediation via concern and trust variable. Notes. Standardized effect sizes for continuous variables; statistical significance levels based on robust standard errors (*** p < 0.001; ** p < 0.01; * p < 0.05); effect in parenthesis is the direct effect of discrimination; indirect effect based on bootstrapping, non-parametric 95% confidence interval in brackets, N = 697; SRMR = 0.00, CD = 0.47. All mediators were allowed to covary with each other. Of these 15 covariance terms, 5 were not significant: Privacy – Solidarity; Privacy –Reciprocated solidarity; Reputation concerns – Government trust; Reputation concerns – Solidarity; Reputation concerns – Reciprocated solidarity.

Fig 1 shows the results of the mediation analysis for DCT support. These partially confirm the patterns from the bivariate analysis, yet some paths do not seem to hold when taking into account confounding variables and the other pathways simultaneously.

In contrast to the bivariate analysis, we do find that ethnic discrimination positively predicts privacy concerns (β = 0.09, *p* = 0.026), and these in turn negatively predict support for DCT (β = −0.15, *p* < 0.001). The indirect effect (discrimination -> privacy -> DCT) is marginally significant (β = −0.01, *p* = 0.058). However, we do not find confirmation for the pathway via increased reputation concerns, since these are neither significantly predicted by discrimination, nor do they significantly predict DCT support (discrimination -> reputation -> DCT: β = −0.00, *p* = 0.72).

Looking at the trust pathway, we observe, as before, a small negative effect of perceived ethnic discrimination on government trust (β = −0.15, *p* < 0.001) and a moderately strong positive effect of government trust on DCT support (β = 0.47, *p* < 0.001). The total indirect effect is β = −0.07 (*p* < 0.001), which is 85% of the total indirect effect of discrimination on DCT. While there is an effect of discrimination such that it erodes generalized trust (β = −0.10, *p* = 0.046), generalized trust does not exert any effect on DCT support above and beyond government trust and the other predictors, resulting in an insignificant indirect pathway (discrimination -> gen. trust -> DCT: β = 0.00, *p* = 0.92).

Lastly, the pathway via solidarity and perceived reciprocated solidarity cannot be confirmed. In the full mediation model, we do not find any evidence that perceived ethnic discrimination predicts solidarity or perceived reciprocated solidarity, nor do the latter affect DCT support, The indirect pathways are not significant (discrimination -> solidarity -> DCT: β = −0.00, *p* = 0.97; discrimination -> rec. solidarity -> DCT: β = 0.00, *p* = 0.47).

Overall, we found a small to moderate total negative indirect effect of perceived ethnic discrimination on DCT support through all mediators (β = −0.08, bootstrapped 95%-CI [−0.13; −0.04]). Thereby, trust in the government emerged as the strongest mediator (85% of the total indirect effect), followed by privacy concerns, which was marginally significant. All remaining mediators were not significant (see Fig 1: The SRMR of 0.00 and Coefficient of Determination (CD) of 0.47 indicate a good model fit.

### DCT opt-in

**Fig 2:**
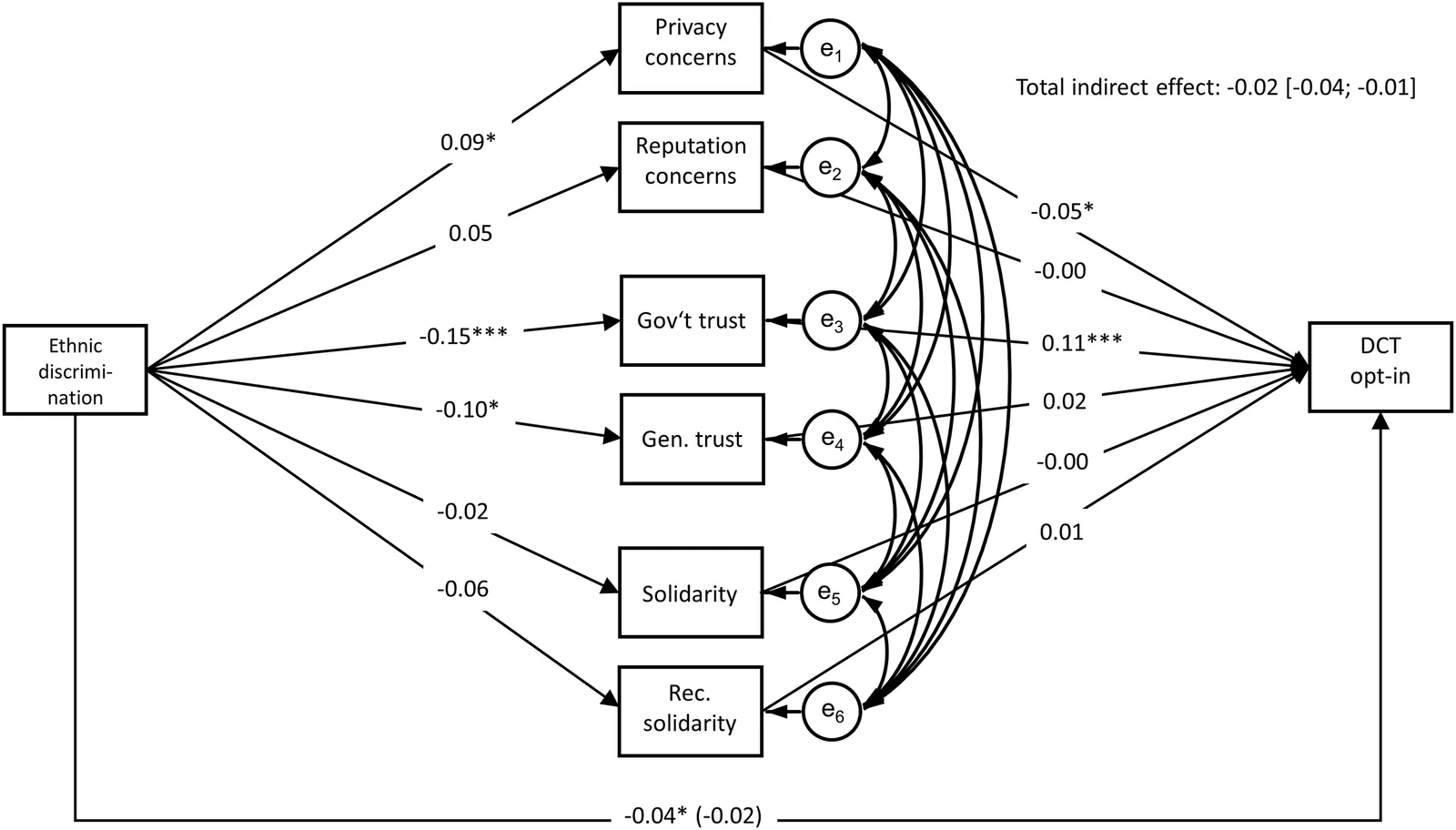
Effect of perceived ethnic discrimination on DCT opt-in, mediation via concern and trust variables. Notes. Standardized effect sizes for continuous variables; statistical significance levels based on robust standard errors (*** p < 0.001; ** p < 0.01; * p < 0.05); effect in parenthesis is the direct effect of discrimination; indirect effect based on bootstrapping, non-parametric 95% confidence interval in brackets, N = 697; SRMR = 0.00, CD = 0.43.

The mediation analysis for DCT opt-in as the dependent variable (Fig 2) generally yields similar patterns as for DCT support, yet only one mediational pathway can be confirmed. The effect paths from discrimination to the mediators are identical to the previous model. With regard to the privacy pathway, we find that privacy concerns, which are increased by perceived discrimination, in turn decrease DCT opt-in by −0.05 (*p* = 0.019). However, the overall estimate for this indirect path is weak and not significant (discrimination -> privacy -> opt-in: β = −0.00, *p* = 0.11). The confirmed pathway runs via the erosion of government trust. Here we find that a one standard deviation increase of government trust is associated with an 11 percentage point increase of participation in DCT. Discrimination decreases opt-in to DCT via reduced government trust by 1.7 percentage points (discrimination -> gov. trust -> opt-in: β = −0.017, *p* < 0.01). All other mediational pathways are not significant. The total negative indirect effect of discrimination on DCT through all mediators is small and significant (β = −0.02, CI[−0.04;-0.01]), but can mostly be attributed to government trust. The SRMR of 0.00 and Coefficient of Determination (CD) of 0.43 nonetheless indicate a good model fit overall.

### Intention of future DCT opt-in among current DCT non-users

In a last step, we examine whether perceived ethnic discrimination will also pose a barrier to future DCT use among those who currently do not use the DCT app (subsample *n* = 425). Specifically, we assess whether discrimination undermines DCT attitudes indirectly through specific relational concerns—i.e., preferences to not reveal personal information towards the government (Government Hide) or other people (People Hide) or the fear that either the government (Government Against) or other people (People Against) will potentially use this information or the DCT app against one’s ethnic or social group.

**Fig 3:**
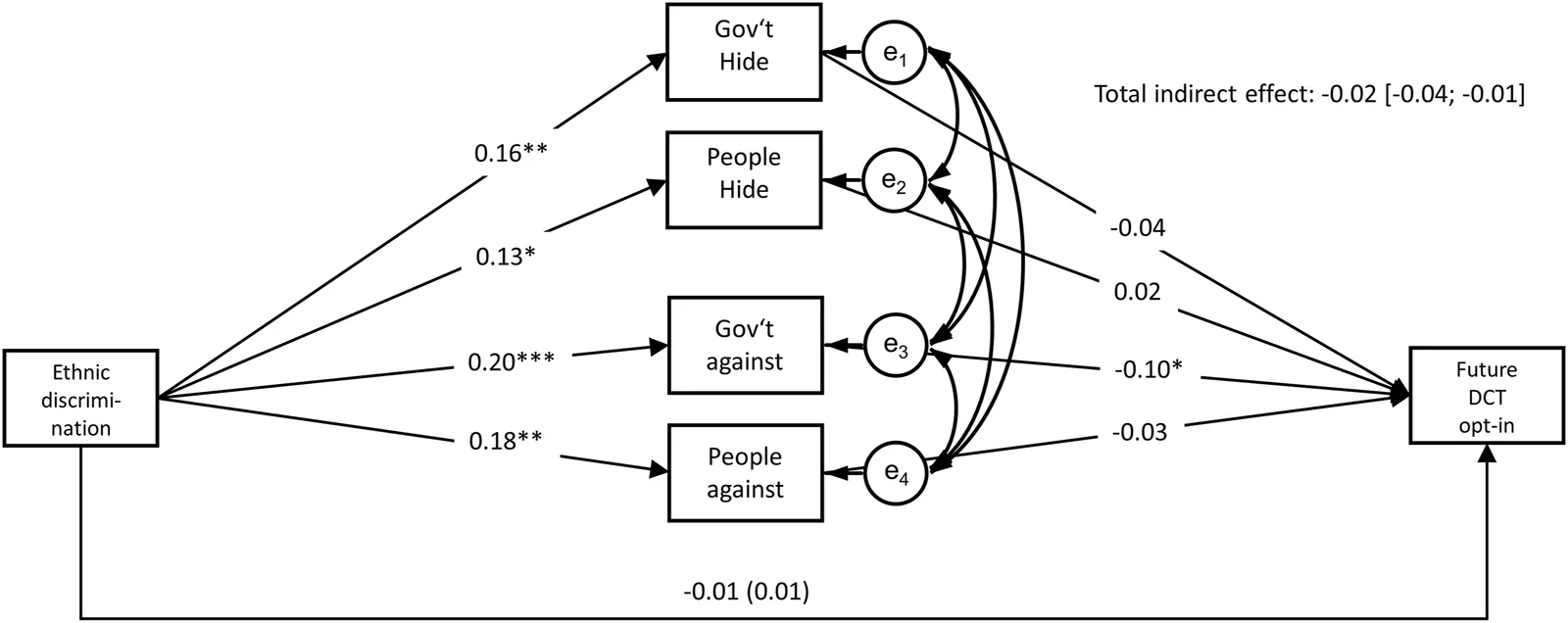
Effect of perceived ethnic discrimination on future DCT opt-in, mediation via relational concern variables in sample of DCT non-users. Notes. Standardized effect sizes for continuous variables; statistical significance levels based on robust standard errors (*** p < 0.001; ** p < 0.01; * p < 0.05); effect in parenthesis is the direct effect of discrimination; indirect effect based on bootstrapping, non-parametric 95% confidence interval in brackets, N = 425; SRMR = 0.00, CD = 0.40.

As the results in Fig 3 show, perceived ethnic discrimination predicts respondents’ fear that the government might use DCT against one’s own ethnic group (β = 0.20, *p* < 0.001). This fear, in turn, negatively predicts future DCT opt-in (β = −0.10, *p* = 0.015), resulting in a small negative indirect effect via this mediator (discrimination -> Government against -> future opt-in: β = −0.02, *p* = 0.034). In addition, ethnic discrimination does predict respondents’ preferences to not disclose information to the government (β = 0.16, *p* < 0.01) or other people (β = 0.13, *p =* 0.019), as well as the fear that other people might exclude members of one’s one group (β = 0.18, *p* < 0.001). However, these factors, in turn, did not significantly predict intentions for DCT opt-in and thus did not mediate the association between discrimination and future opt-in. The bootstrapping results show that the total negative indirect effect of ethnic discrimination via relational concerns is significant (β = −0.02, CI[−0.04; −0.01]).

## Discussion

Digital Contact Tracing (DCT) has been identified as a relatively simple tool that could significantly decrease the time between infection with a virus such as SARS-CoV-2 and an isolation of infected contacts [13] and in combination with self-isolation has proven the by far most efficient strategy to mitigate virus spread [83]. Based on previous research which has shown that trust in the government is a crucial factor for compliance with DCT but also other infection mitigation strategies (see, e.g., [22, 24]), as well as based on the literature examining preventive behavior in racialized minorities [51,84] and the effects of discrimination on racialized groups’ levels of trust [68,85], we suggested that perceived ethnic discrimination might represent a relevant, yet so far neglected factor hampering DCT support. We suggested three mediational pathways which may be underlying this effect: (1) via increasing privacy and reputational concerns, (2) via decreasing trust in government and generalized trust, and (3) via decreasing solidarity and perceived reciprocated solidarity.

Our data generally confirm that experiences of discrimination negatively affect DCT support and DCT use. For attitudes supportive of DCT, the analyses partially corroborated the first (privacy) and the second mediational pathway (trust). Aligning with observations that racialized communities often perceive surveillance technologies as heightened threats due to historical mistrust [70,71], perceived ethnic discrimination indeed indirectly lowered support for DCT through increasing people’s privacy concerns and decreasing trust in the government. Reputation concerns, generalized trust as well as both forms of solidarity did not appear as relevant mediators of the relationship between perceived discrimination and DCT support. Additionally, it is noteworthy that experiences of ethnic and racial discrimination did not decrease people’s solidarity and perceived reciprocated solidarity. This contrasts with the social exclusion theory proposed by Twenge [74], suggesting that while discrimination erodes trust in institutions, it may not necessarily decrease prosociality among citizens in the context of health crises. A plausible explanation for the null-finding regarding solidarity is that for government-administered tools like DCT, trust in the government and health authorities override any considerations of solidarity. The state possesses the technological capacity to potentially misuse the data, which fellow citizens do not have. Hence, prosocial attitudes are likely inhibited by the primary barrier of institutional mistrust. There also was no evidence that individuals with an immigrant background differed overall from those without an immigrant background with regard to any of the crucial explanatory or outcome variables, except for their more frequent experiences of discrimination. Even after taking into account all the proposed mediators as well as a range of control variables we found a direct negative effect of perceived ethnic discrimination on DCT support. A similar pattern was observed for people’s actual participation in DCT. Again, we found evidence that increased privacy concerns and eroded trust in the government together were accounting for the negative relationship between perceived ethnic discrimination and DCT opt-in. Furthermore, we found that trust in the government is the strongest positive predictor, even stronger than effects of perceived own or others’ vulnerability towards COVID-19, indicating that for tools like DCT, trust in the government may outweigh health-related risk perceptions as a driver of collective action This central role of trust aligns with findings from the U.S. of this multi-national study, which similarly identified government trust as a primary mediator for DCT uptake, albeit in the context of cultural value orientations [75].

Lastly, we examined among current DCT non-users whether experiences of discrimination decrease intentions to opt-in to DCT in the future indirectly through specific relational concerns, i.e., preferences to not reveal information towards the government/other people and fears that DCT might be used against their one’s own racialized or social group by the government / other people. We found that ethnic discrimination increased these relational concerns. Yet, only the concern that the government might use DCT against one’s group predicted respondents’ willingness to participate in DCT in the future, hence mediating the relationship between discrimination and willingness for future DCT opt-in.

### Limitations

There are some limitations to our investigation that have to be taken into consideration when interpreting our findings. First, the cross-sectional nature of this study does not allow for any causal interpretations regarding the examined associations. Reverse causality (e.g., low trust increasing perceived discrimination) cannot be fully ruled out. Furthermore, it should be noted that we used a one item measure as a proxy to determine perceived ethnic/racial discrimination. Future studies might employ a more detailed instrument inquiring about a range of subtle to more overt forms of discrimination, which may affect the examined relationships differently (see, e.g., [86]). Moreover, given that discrimination erodes trust over time, future longitudinal research should investigate whether specific policy interventions can effectively reverse this negative impact to foster technology acceptance. Also, even though we found statistically significant associations it should be noted that the effect sizes of some of the examined relationships were small and should hence be interpreted cautiously.

## Conclusion

Our results underline the importance of concerns regarding privacy protection, concerns regarding the use of information against one’s own group as well as trust in government as determinants of compliance with respondent-driven technology-based mitigation measures like DCT. Also, our data draw attention to experiences of ethnic discrimination as a crucial preceding factor increasing privacy and relational concerns as well as eroding government trust, thereby representing a barrier to the implementation of such digital mitigation measures. What follows from these results is that in order to increase people’s willingness to engage in self- and other-protective behaviors to improve infectious disease spread, such as participating in DCT, governments need to address, in the first place, the problem of discrimination. Also, governments should try to re-build trust among individuals who have experienced ethnic discrimination, should ameliorate concerns regarding an abuse of such technology against minority groups can be ruled out and should invest in ensuring citizens’ privacy rights. Various case studies among BAME and BIPOC communities in different settings [70,71] have shown that increased trust-building measures, such as seeking personal contact with minority populations under risk, employing healthcare and administrative personnel speaking the groups’ language [45] and organising targeted focus groups to allow for potential concerns to be directly raised and addressed [46] can be helpful in this regard.

Recommendations for a balanced and critical ethical review of the implementation of COVID apps, their conscious use and ethical pitfalls, including privacy issues [38,87] for their implementation have been outlined [88], and insights gained during the pandemic could also provide an orientation for future scenarios.

Even though our study explicitly focuses on DCT implementation (stage) during the COVID pandemic, we think that our findings might also carry some significance for related problems that societies are facing regarding other strategies that might be considered for preventing the spread of other infectious diseases or for potential future epidemic scenarios.

Taking the evidence from our study and other studies in the field, we conclude that a government that strives to battle discrimination and pursues equal treatment for minority groups will be better equipped to fight a pandemic because the experiences of discrimination, and the resulting loss of trust in society and privacy as well as relational concerns that follow from it reduce compliance with intervention measures.
